# A Case of Endovascular Aortic Repair for Abdominal Aortic Aneurysm with a Saccular Aneurysm in the Severely Angulated Proximal Landing Zone

**DOI:** 10.3400/avd.avd.cr.23-00038

**Published:** 2023-09-30

**Authors:** Akito Imai, Kisato Mitomi, Masataka Sato, Kanji Matsuzaki, Yasunori Watanabe

**Affiliations:** 1 Department of Cardiovascular Surgery, Hitachi General Hospital, Hitachi, Ibaraki, Japan

**Keywords:** EVAR, saccular aneurysm, coil embolization

## Abstract

We report a case of endovascular aortic repair (EVAR) for the abdominal aortic aneurysm complicated by a saccular aneurysm due to a penetrating atherosclerotic ulcer in the severely angulated proximal landing zone. To secure the zone, coil embolization of the saccular aneurysm was performed before stent grafting to treat the abdominal aortic aneurysm. To precisely follow the severely angled proximal neck, we used the Excluder stent-graft system inserted by the body floss technique method instead of the stiff wire method to avoid accordion folding the proximal landing zone. These techniques may expand the indications of EVAR.

## Introduction

Endovascular aortic repair (EVAR) is a minimally invasive treatment for abdominal aortic aneurysm, but the indication for EVAR is limited by unfavorable aortic anatomy including that of a hostile aortic neck.

We report a case of EVAR for the saccular abdominal aortic aneurysm due to a penetrating atherosclerotic ulcer (PAU) in the severely angulated proximal landing zone with using the coil embolization-assisted technique and pairing a flexible stent-graft system with alternative insertion techniques. These techniques may expand the indications of EVAR.

## Case Report

**Figure 1:**
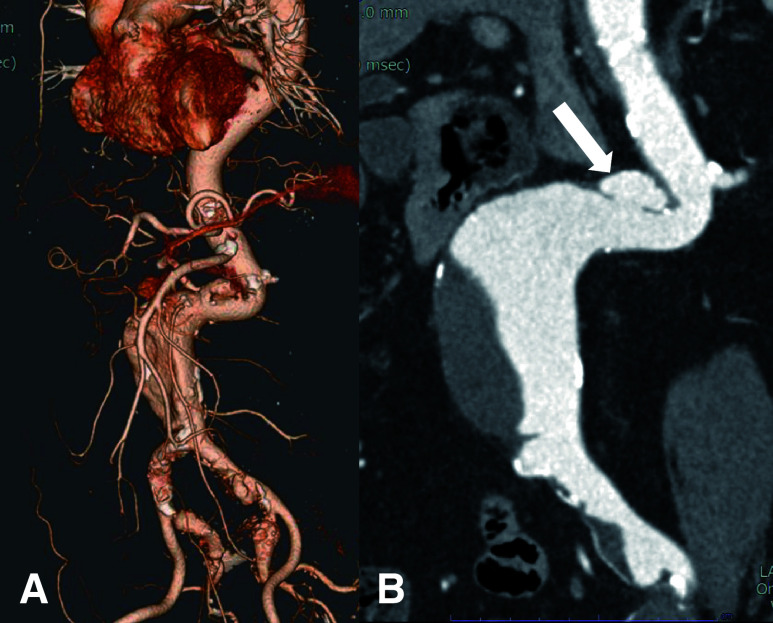
Fig. 1 Preoperative 3-dimensional reconstruction and CTA. (**A**) 3-dimensional reconstruction confirmed infrarenal AAA with severely angulated neck. Aneurysmal dilatation of left common iliac artery and atherosclerotic atheroma of bilateral iliac arteries were observed. (**B**) Preoperative CTA showed the infrarenal AAA with a saccular aneurysm (white arrow) due to a PAU in the proximal landing zone. CTA: computed tomography angiography; AAA: abdominal aortic aneurysm; PAU: penetrating atherosclerotic ulcer.

An 84-year-old woman diagnosed with transverse colon cancer presented with a chief complaint of fecal occult blood. Preoperative multidetector computed tomography revealed an infrarenal abdominal aortic aneurysm (AAA) with a maximum diameter of 50 mm and a severely angulated proximal landing zone (>90°). In addition, a saccular aneurysm due to a PAU was found in the proximal landing zone (**Fig. 1A**).

Past history included hypertension, interstitial pneumonia, and steroids. Due to advanced age, steroid status, and upcoming laparotomy for cancer, we decided on EVAR where a coil embolization of the saccular aneurysm in the proximal landing zone would be performed before stent-graft deployment. We chose the Excluder stent-graft system that our institutional experience has determined as the most flexible. In this case, the PAU ulcer area was localized and intimal continuity was relatively continuous; therefore, we expected that a coil embolization for the saccular aneurysm would secure the proximal landing zone (**Fig. 1B**).

**Figure 2:**
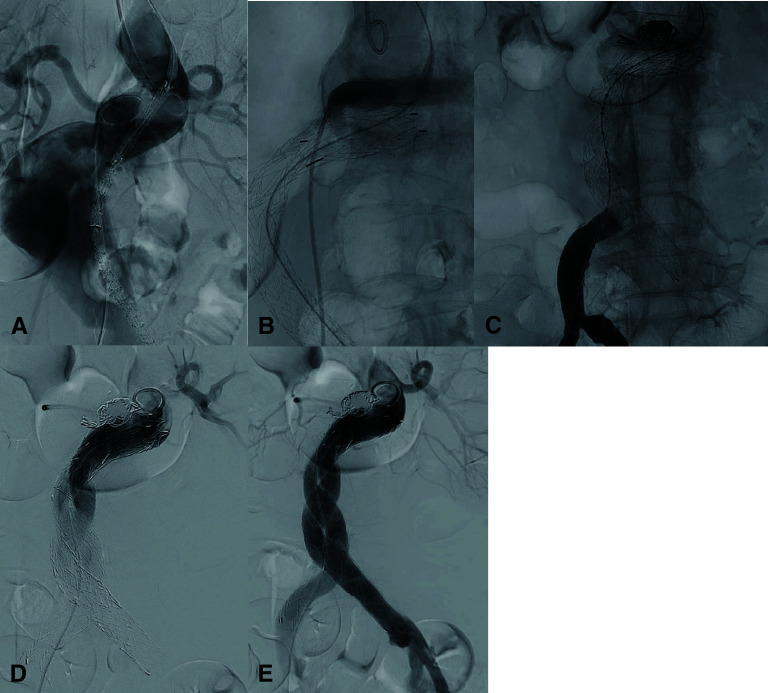
Fig. 2 Angiography during the procedure. (**A** and **B**) The tip of the KMP (Torcon NB®; Cook) catheter introduced from the right femoral artery was placed in a saccular aneurysm of the proximal landing zone. An Excluder main body was deployed below the origin of the lowest renal artery over the tagging wire. (**C**–**E**) Coil embolization of the saccular aneurysm through the KMP catheter confirmed no blood flow inside the aneurysm by aortography. It also showed favorable stent-graft positions without Type 1A endoleak.

**Figure 3:**
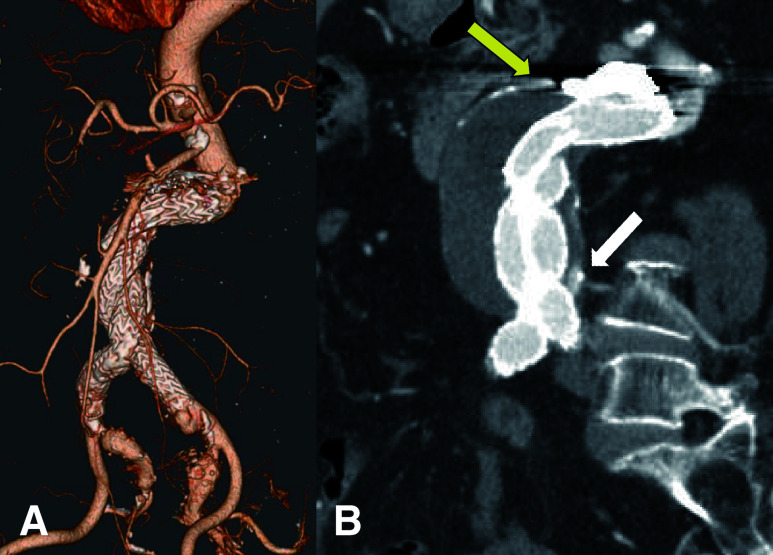
Fig. 3 Postoperative 3-dimensional reconstruction and CTA at 1-year follow-up. (**A**) 3D reconstruction showed favorable stent-graft positions without Type 1A endoleak or leg obstruction. (**B**) Postoperative CTA showed favorable stent-graft positions without Type 1A endoleak (yellow arrow). However, it showed the Type II endoleak from the lumbar artery (white arrow). CTA: computed tomography angiography.

Following general anesthesia, the bilateral femoral artery was exposed. After completion of body floss technique from the right brachial artery to the left femoral artery (Radifocus; Terumo, Tokyo, Japan), a pig-tail catheter was introduced into the suprarenal aorta from the left radial artery (**Fig. 2A**). An 18-Fr DrySeal sheath (W.L. Gore & Associate, Flagstaff, AZ, USA) was introduced from the left femoral artery, while a 9-Fr sheath (Introducer Sheath; Terumo, Tokyo, Japan) was introduced from the right femoral artery. The tip of the KMP (Torcon NB®; Cook, Bloomington, IN, USA) catheter introduced from the right femoral artery was deployed into the saccular aneurysm of the proximal landing zone (**Fig. 2B**). An Excluder RMT261418J aortic main body (W.L. Gore & Associate) was deployed below the origin of the lowest renal artery over the tagging wire (**Figs. 2B** and **2C**). We then performed coil embolization of the saccular aneurysm through the KMP catheter using 10 coils (Tornado 100 mm length; Cook) and confirmed blood flow stoppage inside the aneurysm by aortography (**Fig. 2D**). The ipsilateral leg was successfully deployed with the lower end proximal to the opening of the left internal iliac artery but cannulation of the contralateral limb was abandoned because of the tortuous aortoiliac course. Therefore, the guide wire using the body-floss technique was inserted through the contralateral gate to grasp the wire with an AtrieveTM vascular snare catheter inserted from the right femoral artery. An Exlcuder PLC201400J iliac leg (W.L. Gore & Associate) was sequentially deployed proximal to the opening of the right internal iliac artery. Aortography confirmed complete sealing of the complex AAA with patency of the bilateral renal arteries, external and internal iliac arteries (**Fig. 2E**). Patient recovery was uneventful and discharge occurred on postoperative day 7. Surgery for transverse colon cancer was conducted 52 days after the stent-graft surgery. Follow-up computed tomography angiography (CTA) at postoperative 1 year demonstrated favorable stent-graft positions without Type IA endoleak or migration, but with Type II endoleak from the lumbar artery (**Figs. 3A** and **3B**).

## Discussion

EVAR has been widely used as an alternative to open repair for patients with AAA since it was reported by Parodi et al.^1)^ in 1991. However, the indication for EVAR is limited by unfavorable aortic anatomy including that of a hostile aortic neck. A meta-analysis defined a hostile neck as conditions unsuitable for use of endovascular devices employed in the selected studies, including anatomical factors such as neck length <15 mm, infrarenal neck angulation >60°, neck calcification or thrombus covering >50% of the aortic circumference, and/or a reverse taper morphology.^2)^ We were additionally concerned with the lack of a sufficient proximal landing zone because of the saccular aneurysm due to PAU. Recent studies have reported successful EVAR in hostile necks, but standard anatomical guidelines for EVAR selection are scarce.^3,4)^ In our case, the advanced age, long-term steroid use, and upcoming laparotomy for cancer made EVAR attractive despite a severely angulated neck and a saccular aneurysm in the proximal landing zone.

We previously reported a coil embolization-assisted thoracic endovascular aortic repair (TEVAR) for a saccular descending aortic aneurysm with an insufficient distal landing zone.^5)^ Here, we referred to a similar cerebral technique^6)^ where TEVAR could be adapted to a short distal landing zone by performing coil embolization of the celiac artery and the saccular aneurysm. In this technique, the coil embolization fills the saccular aneurysm but does not use to embolize the endoleak, resulting in an enlarged landing zone. We were therefore able to completely embolize the saccular aneurysm with the coils, and the newly created landing zone allowed successfully deployment of the stent graft without Type IA endoleak.

To the extent of our awareness, no direct comparisons of different stent graft types for EVAR for AAA are reported and optimal selection of stent graft type for severely angulated necks remains problematic.^7)^ Smeds et al. reported in a retrospective review that the C3 Excluder stent-graft system for an inadequate proximal landing zone resulted in favorable short-term outcomes and significantly reduced the addition of proximal extension cuffs.^8)^ Furthermore, Zeng et al. reported an EVAR case with a severely angulated proximal neck and hostile access treated successfully with an Excluder stent-graft system.^9)^ Bastos et al. also reported acceptable early results with a severely angulated proximal landing zone using an Endurant stent-graft system, with no loss of sealing length in exceedingly angulated cases.^10)^ In addition, Albertini et al. demonstrated acceptable mid-term results with endovascular repair using an Aorfix stent-graft system in patients with severely angulated necks.^4)^ Recently, Morikage et al. reported the reverse slider technique using the Endurant stent-graft system.^11)^ This is a method of securing central sealing by pushing up the graft edge while placing it, and it is thought that sealing is possible even with a neck length of 10 mm. Our choice of the Excluder stent-graft system was based on previous experiences and anecdotal evidence from our center where we find it to be the most flexible and adaptable within the blood vessel, resulting in a better outcome. The stent-graft system is usually inserted through a stiff guide wire, and in cases with severely angulated proximal necks, the insertion of the stiff wire changes the native vessel creating longitudinal folds or aortic “accordion folds.” The creation of these accordion folds in the vessel also tends to result in a poor landing zone seal after deployment. Therefore, we find the technique of employing the body-floss technique with the Excluder stent-graft system preferable so that vascular insertion does not force distortions creating the accordion folds of the native vessel during deployment. It is the brachiofemoral through-and-through wire technique and it is used to track endovascular devices across tortuous aorto-iliac anatomy encountered during endovascular repair of abdominal or thoracic aortic aneurysms. This method allows the device to be placed in a manner that follows the course of the anatomical vessels.^12,13)^ Furthermore, the main body approach was via the left femoral artery so that the device would retain a C shape. After deploying the contralateral leg, pushing up the device then better sealed the severely angulated neck to the greater curvature side. Intraoperative aortography and 1-year follow-up CTA demonstrated a complete sealing of the AAA without Type IA endoleak and migration but Type II endoleak from the lumbar artery will require life-long vigilance.

## Conclusion

This coil embolization-assisted technique may expand the utility of EVAR when a saccular aneurysm has an insufficient proximal landing zone. Furthermore, choosing a flexible stent-graft system (such as Excluder) and using alternative inserting techniques, such as the body floss technique, ensures good sealing even in severely angulated proximal necks.

## Informed Consent

The informed consent has been obtained from the patient and participants.

## Disclosure Statement

The authors have no conflicts of interest to declare.

## Author Contributions

Study conception: AI

Data collection: AI

Investigation: AI

Writing: AI

Critical reviews and revision: all authors

Final approval of the article: all authors

Accountability for all aspects of the work: all authors.
